# Development of a colloidal gold-based lateral flow dipstick immunoassay for rapid qualitative and semi-quantitative analysis of artesunate and dihydroartemisinin

**DOI:** 10.1186/1475-2875-13-127

**Published:** 2014-03-31

**Authors:** Lishan He, Tiegui Nan, Yongliang Cui, Suqin Guo, Wei Zhang, Rui Zhang, Guiyu Tan, Baomin Wang, Liwang Cui

**Affiliations:** 1 College of Agronomy and Biotechnology, China Agricultural University, Beijing, People’s Republic of China; 2 State Key Laboratory of Dao-di Herbs, National Resource Center for Chinese Materia Medica, China Academy of Chinese Medical Sciences, Beijing 100700, People’s Republic of China; 3 Department of Entomology, The Pennsylvania State University, University Park, Pennsylvania, USA

**Keywords:** Dipstick, Artemisinin, Artesunate, Dihydroartemisinin, Antimalarial

## Abstract

**Background:**

Artemisinin-based combination therapy (ACT) plays an indispensable role in malaria control and elimination. However, the circulation of counterfeit, substandard drugs has greatly threatened malaria elimination campaigns. Most methods for the analysis of artemisinin and its derivatives require expensive equipment and sophisticated instrumentation. A convenient, easy-to-use diagnostic device for rapid evaluation of the quality of artemisinin drugs at the point-of-care is still lacking. In this study a lateral flow dipstick immunoassay was developed for qualitative and semi-quantitative analysis of artesunate (ATS) and dihydroartemisinin (DHA) in anti-malarial drugs.

**Methods:**

This assay was based on a monoclonal antibody (mAb) raised against ATS. ATS-bovine serum albumin and goat anti-mouse IgG, used as the test capture reagent and the control capture reagent, were coated on the nitrocellulose membrane to form the test line and control line, respectively. The conjugate pad was saturated with the gold-labelled anti-ATS mAb.

**Results:**

The indicator range of the dipsticks, defined as lowest concentration of the target analytes between which the test line was not visible, were 100-200 and 200-500 ng mL^-1^ for ATS and DHA, respectively. No competitive inhibition was observed up to 5,000 ng mL^-1^ of quinine, chloroquine diphosphate salt, primaquine phosphate, pyrimethamine, lumefantrine, amodiaquine, piperaquine tetraphosphate tetrahydrate or pyronaridine tetraphosphate. Semi-quantitative analysis of ATS and DHA in commercial drugs and raw drug materials with the dipsticks produced result agreeable with those determined by high performance liquid chromatography (HPLC). Storage test showed that the indicator range for artemisinins remained unchanged after a week at 37°C and increased four-folds after six months of storage at 4°C or ambient temperature.

**Conclusions:**

The new selected mAb 3D_8_2G_7_ with high avidity and broad cross reactivity for artemisinins was used to develop and optimize a dipstick immunoassay for qualitative and semi-quantitative analysis of ATS and DHA in anti-malarial drugs. The semi-quantitative analysis of ATS and DHA in commercial drugs and raw drug materials, and the specificity test of the artemisinin-related drugs both proved the accurate performance of the developed dipsticks for semi-quantitation of ACT samples. The dipstick may be used as a point-of-care device for identifying substandard and counterfeit ATS- and DHA-containing anti-malarial drugs.

## Background

Artemisinin-based combination therapy (ACT) plays an indispensable role in malaria control and elimination. However, counterfeit and substandard drugs greatly threaten malaria elimination campaigns. This problem is particularly widespread in resource-poor developing countries [1]. Five studies of artemisinin drugs from countries in Southeast Asia, found that 43% of samples failed chemical assay analysis, while 42% of samples failed packaging tests [2-6]. A survey conducted in 2006 in Thailand revealed that 15.4% of artesunate (ATS), 11.1% of chloroquine, and 29.4% of quinine were substandard [7]. While this problem is serious in some Southeast Asian countries, some African countries, where malaria is most prevalent, may be similarly worrisome. Investigations on the quality of artemisinin derivatives in Kenya and DR Congo detected the circulation of counterfeit, substandard drugs [8]. A wider survey in six most severely malarious parts of Africa also found that significant proportions of the anti-malarial drugs, including artemisinin derivatives, failed the content and dissolution tests [9]. A recent review of anti-malarial drug qualities in Southeast Asia and sub-Saharan Africa, which shows that at least 35% of the anti-malarials failed the chemical analysis and large proportions of them as counterfeit drugs, clearly underline the severity of the fake and substandard anti-malarial drug situation [10].

Fake and substandard drugs not only reduce the treatment efficacy and promote resistance development, but also may result in life-threatening complications and even deaths of the patients [11]. The progression of malaria from mild to severe disease is rapid, especially in young children, giving drugs that contain little or no active ingredients is parallel to “manslaughter” [11]. As counterfeit or substandard anti-malarials imperil the great stride made towards malaria control in the recent years, there is an urgent need to strengthen quality control of anti-malarial drugs.

Most methods for the analysis of artemisinin and its derivatives require expensive equipment and sophisticated instrumentation. In the recent years, some rapid and more economic methods for quality surveys of anti-malarials have been developed. Those include fast red TR [12], thin-layer chromatography [3,9], Fourier-transform infrared imaging and Raman spectroscopy [13-15], and near-infrared spectroscopy [16]. Yet, a convenient, easy-to-use diagnostic device for rapid evaluation of the quality of artemisinin derivatives at the point-of-care is still lacking. Given that malaria-endemic populations are very familiar with the dipstick-type of malaria rapid diagnostic tests, the aim of this study is to develop a lateral flow dipstick for qualitative and semi-quantitative detection of artemisinins in anti-malarial drugs. The dipsticks are a one-step assay with minimum handling of reagents, and the results are readily read by naked eyes [17]. To develop such a dipstick assay, the antibody is used as the core reagent. Our laboratory has obtained a hybridoma cell line that secreted a monoclonal antibody (mAb) 3H_2_ against ATS, and developed an indirect competitive ELISA (icELISA) [18]. Although the mAb 3H_2_ was specific for artemisinins, its low antibody titer was not suitable for dipstick development. After re-selection of a hybridoma library, a mAb with high avidity and broad cross reactivity for artemisinins was identified and further used to develop and optimize a dipstick immunoassay for qualitative and semi-quantitative analysis of ATS and dihydroartemisinin (DHA) in anti-malarial drugs.

## Methods

### Reagents

Details of the reagents used in this study are provided in Additional file 1: Table S1.

### Production of anti-ATS mAb

ATS chosen as the hapten was conjugated to ovalbumin (OVA) via the previously described active ester method [18]. The protein-hapten conjugates were used to immunize mice, which were used for hybridoma production. Spleen cells collected from the immunized mouse were fused with murine SP2/0 myeloma cells at a ratio of 10:1. The hybridomas were selected in complete medium [Dulbecco’s modified Eagle’s medium (DMEM) supplemented with 20% fetal bovine serum (FBS), 0.2 M glutamine, 50,000 U L^-1^ penicillin, and 50 mg L^-1^ streptomycin with 1% (v/v) thymidine] for approximately two weeks. Monitoring of the titer of supernatants or mAbs, and screening of positive hybridoma clones were done by indirect ELISA (iELISA). The specificity and sensitivity of supernatant and mAbs was evaluated by icELISA. The protocol for iELISA and icELISA was the same as that described previously [18,19]. Positive hybridomas which produced antibodies having cross reactivity with artemisinin, ATS, DHA and artemether (ATM) were selected and cloned twice by limiting dilution, followed by expansion for large-scale production of mAbs. The titer of the antibody was defined as the fold dilution giving an absorbance of 1.0 in iELISA. The mAb with a high titer and showing broad cross reactivity against artemisinin derivatives was selected for dipstick development.

### Development of colloidal gold-based lateral-flow dipstick immunoassay

For the preparation of the nitrocellulose membrane, ATS-bovine serum albumin (BSA) and goat anti-mouse IgG were used as a test capture reagent and control capture reagent, respectively. Both the test and control capture reagents were dispensed separately as lines at the bottom and top of the membrane strips (300 × 25 mm) using a dispenser. The distance between the lines was 0.5 cm. After dispensing, the membrane was dried at 37°C for 30-60 min to immobilize the reagents.

Colloidal gold with an average particle diameter of 30 nm (G30) was prepared according to the procedure described by Frens [20]. The colloidal gold solution was adjusted to pH 7.8 with potassium carbonate solution while stirring in a 10 mL beaker. For antibody coating, 40 μL of the mAb aqueous solution at a concentration of 2.5 mg mL^-1^ was added to the colloidal gold solution. After coating at room temperature for 20 min, the gold-antibody suspension was further stabilized by adding 200 μL of 10% (w/v) BSA. The mixture was stirred for 15 min, and then let sit for 5 min, followed by centrifugation at 9500 rpm for 40 min. The supernatant was then carefully discarded, and the obtained gold-antibody conjugate was brought to 1 mL with the GBA^+^ buffer. Finally, the conjugate pad was saturated with the gold-antibody conjugate, and dried at 37°C overnight.

**Figure 1:**
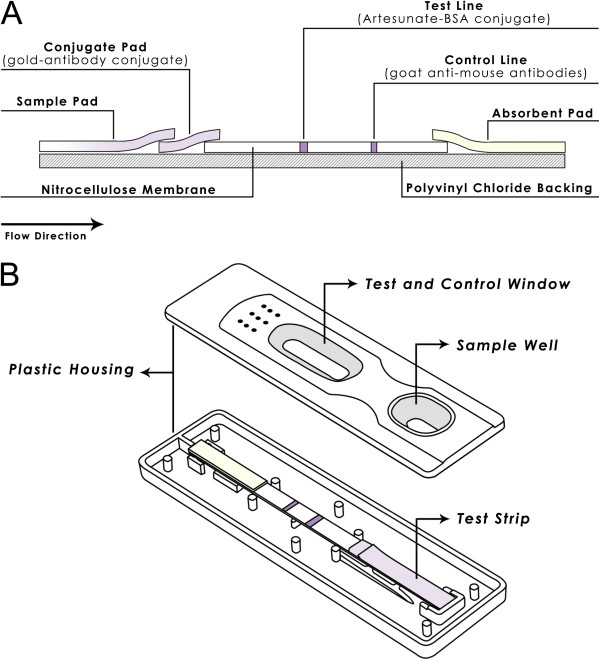
Assembly of colloidal gold-based dipstick (A) and plastic housing (B) where a dipstick is mounted.

The dipstick was assembled as shown in Figure 1A. The dipstick consisted of a polyvinyl chloride (PVC) backing on which a nitrocellulose membrane, conjugate pad, sample pad and absorbent pad were pasted. The sample pad was attached to the bottom of the dipstick (at the origin of the sample flow), with 1-2 mm overlapping with the conjugate pad. The conjugate pad was attached to the bottom of the membrane with 1-2 mm overlapping with the membrane. The absorbent pad was attached to the top of the membrane in a similar manner as the conjugate and sample pads. After complete assembly, the entire plastic backing-polyester plate was further cut into strips (60 × 4 mm). The individual strip was mounted in a plastic housing (Figure 1B) and sealed in aluminum foil pouch until use.

### Evaluation of the dipsticks: indicator range and cross reactivity

**Figure 2:**
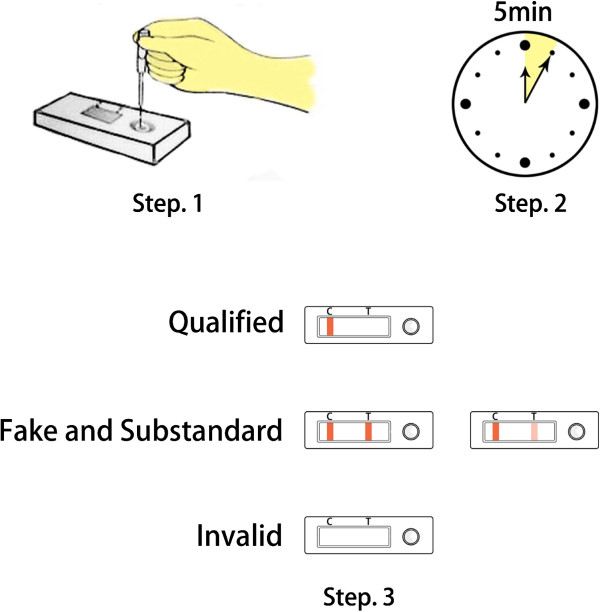
A schematic of the assay steps.

Stock solutions of artemisinin, ATS or DHA were prepared in acetonitrile at 2 mg mL^-1^. The standard solutions were 200, 500, 1,000, 2,000, 5,000, 10,000 and 20,000 ng mL^-1^ of artemisinin, 10, 20, 50, 100, 200, 500 and 1,000 ng mL^-1^ of ATS and 20, 50, 100, 200, 500, 1,000 and 2,000 ng mL^-1^ of DHA in distilled water. The indicator range, defined as the lowest concentration of the target analytes between which the test line was not visible, is used to indicate the sensitivity of the dipstick. To estimate the indicator range of the dipsticks, 80 μL of standard solutions were added dropwise into the sample well. The colour of the test and control line was visually observed within 5 min (Figure 2). Quinine, chloroquine diphosphate salt, primaquine phosphate, pyrimethamine, lumefantrine, amodiaquine, piperaquine tetraphosphate tetrahydrate and pyronaridine tetraphosphate were tested for specificity. The stock solutions of these eight drugs at 1 mg mL^-1^ were prepared in absolute ethanol (quinine), water (chloroquine diphosphate salt, primaquine phosphate, amodiaquine, or pyronaridine tetraphosphate), acetonitrile (pyrimethamine or piperaquine tetraphosphate tetrahydrate), and chloroform (lumefantrine). Final water dilutions of these stock solutions were used at 5,000 ng mL^-1^. Each sample was analyzed in triplicates.

### Testing of commercial artemisinin-based drugs

All commercial drugs were purchased from regular medical establishments and legitimate pharmacies. These include ATS tablets (Lot no. 040502, AS100801, ATS 50 mg per pill, Guilin pharmaceutical CO., Ltd), ATS for injection (Lot no. LA110102, ATS 60 mg per tube, Kunming Pharma Corp.), Artesun-Plus (Lot no. SH120707, 100 mg per pill, Guilin pharmaceutical CO., Ltd), Artecospe (Lot no.S130304, 100 mg per pill, Guilin pharmaceutical CO., Ltd), and DHA and Piperaquine Phosphate tablets (Lot no. 030211, 010710, 020807, DHA 40 mg per pill, Chongqing Holley Healthpro Pharmaceutical CO., Ltd). Anti-malarial drug tablets were grounded in a clean mortar to a fine powder. The powdered drugs were transferred into a 15 mL tube and extracted with 1.5 mL of acetonitrile. The acetonitrile extract was sonicated in a Branson SB5200 ultrasonic oscillation under room temperature for 30 min, followed by centrifugation at 2,080 × g for 30 min. The extraction procedure was repeated three times and the supernatants were combined and filtrated through a 0.5 μm syringe filter. The filtrates were collected and stored at 4°C before analysis. For the commercial samples, the sample extracts were diluted into 2 mg mL^-1^ with acetonitrile as stock solutions for the dipstick and high performance liquid chromatography (HPLC) assays based on the labelled content of the commercial drugs. The active pharmaceutical ingredient (API) of ATS and DHA were dissolved with acetonitrile at a concentration of 2 mg mL^-1^ as stock solutions. All stocks were then diluted with distilled water to concentrations in the working range of the dipstick. Each sample was analyzed in triplicates.

### HPLC method

The HPLC system consisted of a Waters 600E multisolvent delivery system and a Waters 2487 dual λ absorbance detector (Milford, MA, USA). The mobile phase, standards, drug and sample extracts obtained above were filtered through a 0.5 μm syringe filter prior to HPLC. A C18 column (250 × 4.6 mm, 5 μm particle size; Thermo, Vantaa, Finland) was used to resolve ATS and DHA. A mobile phase for ATS was a mixture of 60% acetonitrile and 40% ultra pure water at a flow rate of 1 mL min^-1^, while the mobile phase for DHA was 40% acetonitrile and 60% ultra pure water at a flow rate of 1 mL min^-1^. The injection volume was 20 μL. Detection wavelength was 210 nm [21].

### Storage test of the dipsticks

To determine the performance of the dipsticks after storage, the dipsticks were stored for one week at 37°C, and for 6 months either at 4°C or ambient temperature. After storage, their indicator ranges for artemisinin, ATS and DHA was re-evaluated.

## Results

### Production of anti-ATS mAb

**Figure 3:**
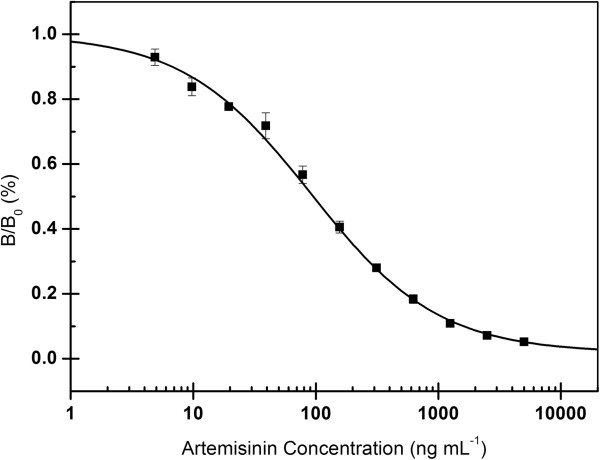
**Standard inhibition curve of artemisinin in icELISA format.** B_0_ and B are absorbance in the absence and presence of competitors, respectively. Concentration causing 50% inhibition by artemisinin was 96 ng mL^-1^. Each value represents the mean of three replicates.

Screening of the hybridoma library identified nine clones secreting anti-ATS mAbs. The titer, sensitivity and specificity of these mAbs were evaluated utilizing icELISA. The mAb designated as 3D_8_2G_7_ showed the highest titer of 3.2 × 10^4^. The 3D_8_2G_7_ mAb showed strong reactivity with ATS (458%), DHA (325%) and artemisinin (100%), but significantly lower cross reactivity with ATM (1.5%), (Table 1). The concentration causing 50% of inhibition (IC_50_) and the working range in icELISA for artemisinin was 96 ng mL^-1^, and 17-544 ng mL^-1^ (Figure 3). Therefore, the mAb 3D_8_2G_7_ was selected to develop a colloidal gold-based lateral-flow dipstick immunoassay.

**Table 1:** Cross reactivity of artemisinin and related analytes on icELISA

**Analytes**	**IC**_**50 **_**(ng/mL)**	**Cross-reactivity**^**a **^**(%)**
Artemisinin	96 ± 13^b^	100
ATS	21 ± 1	460 ± 86
DHA	30 ± 3	330 ± 35
ATM	6620 ± 450	1 ± 0.2

^a^Cross-reactivity (%) = (IC_50_ of artemisinin/IC_50_ of other compound) × 100.

^b^Data represent means of triplicates ± SD.

ATS, artesunate; DHA, dihydroartemisinin; ATM, artemether.

### Development of colloidal gold-based lateral-flow dipstick immunoassay

**Figure 4:**
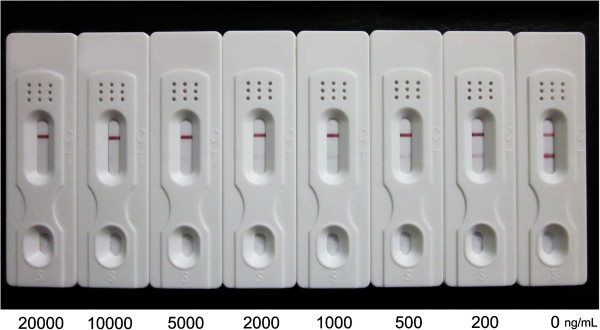
**Dipsticks showing colour changes corresponding to concentrations of artemisinin in distilled water.** The indicator range was 2,000-5,000 ng mL^-1^ for artemisinin. Each sample dilution was analyzed in triplicates and the figure shows the representative pictures.

**Figure 5:**
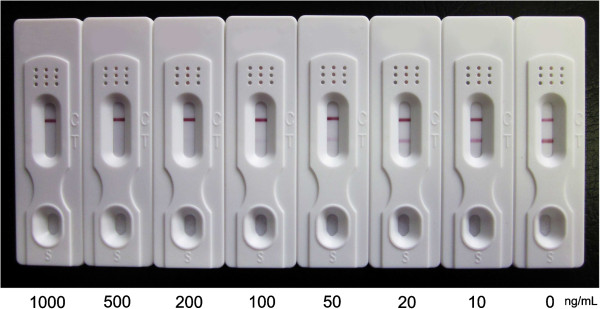
**Dipsticks showing colour changes corresponding to concentrations of ATS in distilled water.** The indicator range was 100-200 ng mL^-1^ for ATS. Each sample dilution was analyzed in triplicates and the figure shows the representative pictures.

**Figure 6:**
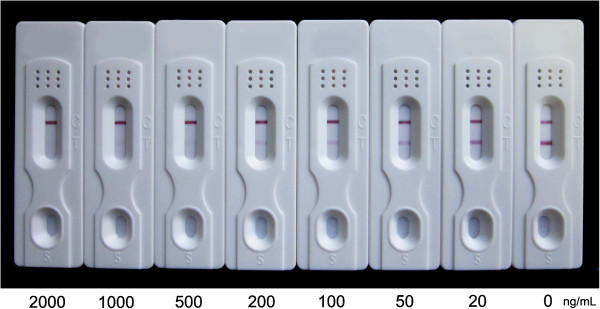
**Dipsticks showing colour changes corresponding to concentrations of DHA in distilled water.** The indicator range was 200-500 ng mL^-1^ for DHA. Each sample dilution was analyzed in triplicates and the figure shows the representative pictures.

Since the sensitivity of the dipstick assay is based on visual evaluation of the colour intensity of the test and control lines, the concentrations of ATS-BSA conjugate and goat anti-mouse antibody coated on the membrane and the amounts of colloidal gold-mAb were first optimized in order to obtain the lowest indicator range for artemisinin derivatives. The final, optimum concentrations of ATS-BSA, goat anti-mouse antibody and colloidal gold-mAb 3D_8_2G_7_ were 2 mg mL^-1^, 2 mg mL^-1^ and 0.1 mg mL^-1^, respectively. The concentration-dependent colour intensity of the dipsticks for standard artemisinin, ATS and DHA in distilled water are illustrated in Figures 4, 5 and 6. The indicator range were 2,000-5,000 ng mL^-1^, 100-200 ng mL^-1^ and 200-500 ng mL^-1^ for artemisinin, ATS and DHA, respectively.

**Figure 7:**
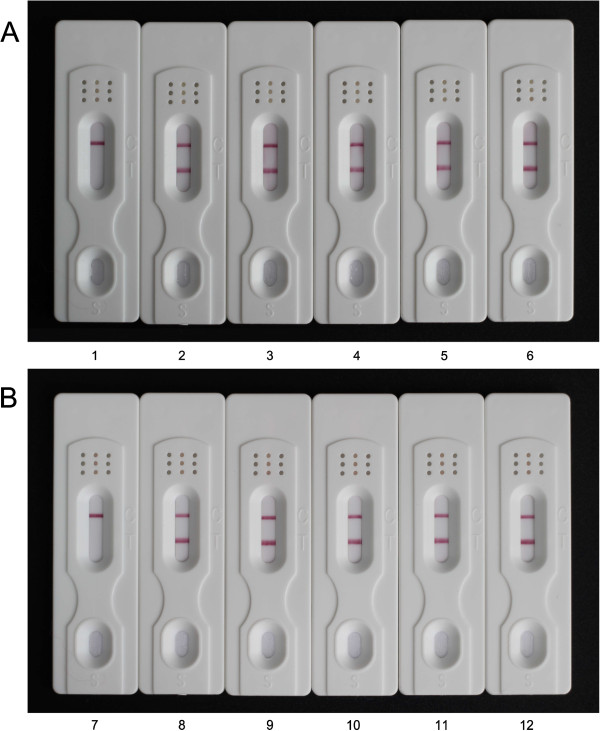
**Dipstick specificity tests of commonly used anti-malarial drugs (A and B).** Each standard solution (1 mg mL^-1^) was diluted in distilled water at a concentration of 5,000 ng mL^-1^. (1) artemisinin; (2) quinine; (3) chloroquine diphosphate salt; (4) primaquine phosphate; (5) pyrimethamin; (6) blank; (7) artemisinin; (8) lumefantrine; (9) amodiaquine; (10) piperaquine tetraphosphate tetrahydrate; (11) pyronaridine tetraphosphate and (12) blank. Each sample was analyzed in triplicates and the figure shows the representative pictures.

To determine the specificity of the dipstick for the artemisinin family drugs, other commonly used anti-malarial drugs were used for the evaluation of cross reactivity. No inhibition was observed for quinine, chloroquine diphosphate salt, primaquine phosphate, pyrimethamine, lumefantrine, amodiaquine, piperaquine tetraphosphate tetrahydrate or pyronaridine tetraphosphate at a concentration of 5,000 ng mL^-1^, whereas the test line was completely inhibited by artemisinin (Figure 7A, B), suggesting that this dipstick had no cross reactivity with other commonly used anti-malarial drugs, some of which are the partner drugs used in ACT.

### Semi-quantitative analysis of API of ATS and DHA

The contents of ATS and DHA API purchased from online stores were examined with the dipsticks using several dilutions. The stock solutions of ATS were diluted 5,000-, 10,000-, 20,000- and 25,000-fold to obtain predicted concentration of 400, 200, 100 and 80 ng mL^-1^, respectively. Test lines for ATS API purchased from store A and B were not detectable after 5,000-fold dilution, whereas they showed faint colour at 1:10000 for the ATS API from store A and 1:20000 for the ATS API from store B. This result indicated that the ATS concentration from store A was between 1-2 mg mL^-1^ and the ATS concentrations from store B was between 2-4 mg mL^-1^, which meant that the determined value to the actual content was 60-120% for API A and 94-188% for API B. The ATS contents from the two stores were also determined by HPLC and the results agreed well with those of the dipstick assay (Table 2).

**Table 2:** Concentration of API of ATS and DHA determined with dipsticks and HPLC

**Ingredient**	**Store no.**	**Theoretical a.i. content**^**a **^**(mg/mL)**	**Measured content by dipsticks (mg/mL)**	**Measured content by HPLC**^**b **^**(mg/mL)**
ATS	A	2.00	1-2	1.67 ± 0.01
	B	2.00	2-4	2.13 ± 0.01
DHA	A	2.00	0.8-2	1.30 ± 0.03
	B	2.00	0.8-2	1.24 ± 0.01
	C	2.00	0	0

^a^Theoretical content of active ingredients was base on the process dissolved 50 mg of API in acetonitrile and diluted with acetonitrile to 25 mL;

^b^Data represent means of triplicates ± SD.

API, active pharmaceutical ingredient; ATS, artesunate; DHA, dihydroartemisinin.

Three DHA APIs purchased from three stores were similarly evaluated using both dipstick and HPLC assays. These DHA samples were first dissolved in acetonitrile at a concentration of 2 mg mL^-1^, then diluted 1,000-, 2,000- and 4,000-fold with distilled water to obtained serial theoretical concentrations of 2,000, 1,000 and 500 ng mL^-1^. The test lines for DHA API from store A and B showed no colour at 1,000- and 2,000-fold dilution, respectively, whereas the colour began to show after 4,000-fold dilutions, suggesting that their DHA concentrations were 0.8-2 mg mL^-1^ (i.e., the determined values were 62-154% for API A and 65-161% for API B to the actual content). These results also agreed with the HPLC result (Table 2). In contrast, a DHA sample purchased from store C could only be partially dissolved in acetonitrile. When testing on the dipstick at the predicted concentrations, no colour inhibition was observed for the test lines, suggesting that the DHA content was lower than the indicator range of the dipstick. HPLC analysis of this sample revealed that the sample from store C was a counterfeit sample and contained no DHA.

### Semi-quantitative analysis of artemisinin-based anti-malarial drugs

Concentrations of active ingredients in the stock solutions of artemisinin-based anti-malarial drugs were determined using the dipsticks. The ATS sample stocks [ATS tablets (Lot no. 040502, Lot no. AS100801), Artesun-Plus (Lot no. SH120707), Artecospe (Lot no.S130304) and ATS for injection (Lot no. LA110102)] were diluted in distilled water for 5,000, 10,000, 20,000, 30,000 and 40,000 times, which gave anticipated concentrations of 400, 200, 100, 66.7 and 50 ng mL^-1^. When analysed using the dipsticks, no colour development was observed on the test line for the 5,000- and 10,000-fold dilutions, whereas the intensity of the colour of test line for the 20,000-, 30,000-, 40,000-fold dilutions gradually increased. This suggested that the ATS concentration in all 20,000-fold diluted samples was lower than the indicator range, which indicated that the ATS concentration in the stock solution was between 2-4 mg mL^-1^ (100-200% to the actual content). This result agreed with the result determined by HPLC (Table 3).

**Table 3:** Concentration of ATS and DHA in commercial drugs determined with dipsticks and HPLC

**Drug names**	**Lot no.**	**The claimed content (mg/pill, tube)**	**Theoretical a.i. content**^**a **^**(mg/mL)**	**Measured content by dipsticks (mg/mL)**	**Measured content by HPLC**^**b **^**(mg/mL)**
ATS tablets	040502	50	2.00	2-4	2.33 ± 0.00^c^
	AS100801	50	2.00	2-4	2.12 ± 0.01^c^
ATS for injection	LA110102	60	2.00	2-4	2.12 ± 0.01^c^
DHA and piperaquine phosphate tablets	030211	40	2.00	1-2.5	2.04 ± 0.03^c^
	010710	40	2.00	1-2.5	2.03 ± 0.01^c^
	020807	40	2.00	1-2.5	2.02 ± 0.00^c^
Artesun-Plus	SH120707	100	2.00	2-4	2.21 ± 0.01
Artecospe	S130304	100	2.00	2-4	2.08 ± 0.01

^a^Theoretical content of active ingredients according to the values shown on the label;

^b^Data represent means of triplicates ± SD.

^c^Data from Wang et al. 2012.

Stock solutions of the DHA and piperaquine phosphate tablets (Lot no. 030211, 010710, 020807) were diluted 2,000, 4,000, 5,000, 10,000, and 20,000 times, and the theoretical concentration after serial dilutions were 1,000, 500, 250, 200 and 100 ng mL^-1^. The test line of the 5,000-fold dilution began to develop a faint colour, suggesting that the DHA concentrations in the solutions were 1-2.5 mg mL^-1^ (50-125% to actual content). The DHA concentrations in the solutions determined by HPLC agreed well with those by dipsticks (Table 3).

### Stability of the dipsticks after storage

The shelf life of the dipsticks was evaluated under three storage conditions. There was no change in the sensitivity of the dipsticks after a week at 37°C. After three months of storage at 4°C and ambient temperature, the indicator range of the dipsticks for artemisinin, ATS and DHA increased to 5,000-10,000, 200-500 and 1,000-2,000 ng mL^-1^, respectively. The indicator range was also increased for these artemisinin family compounds after storage for six months at 4°C and ambient temperature (Table 4).

**Table 4:** The storage stability of the dipsticks

**Analytes**	**Storage conditions**	**Indicator range (ng/mL)**			
		**Storage time**			
		**0 day**	**One week**	**3 months**	**6 months**
Artemisinin	4°C	2,000-5,000	2,000-5,000	5,000-10,000	10,000-20,000
	Ambient temperature	2,000-5,000	2,000-5,000	5,000-10,000	10,000-20,000
	37°C	2,000-5,000	2,000-5,000	-	-
ATS	4°C	100-200	100-200	200-500	500-1,000
	Ambient temperature	100-200	100-200	200-500	500-1,000
	37°C	100-200	100-200	-	-
DHA	4°C	200-500	200-500	1,000-2,000	1,000-2,000
	Ambient temperature	200-500	200-500	1,000-2,000	1,000-2,000
	37°C	200-500	200-500	-	-

## Discussion

A simple dipstick assay was developed for qualitative and semi-quantitative analysis of contents of active ingredients in the artemisinin family anti-malarials, except ATM. When artemisinin, ATS or DHA is present in the testing drugs at the concentrations above the indicator ranges for these compounds, the test line will disappear. If serially diluted samples are used, this would give us a semi-quantitative result. As water solubility of some artemisinin derivatives is low, the stock solutions need to be prepared in DMSO or acetonitrile. Alternatively, to avoid problems with the handling of acetonitrile, methanol and acidified methanol could be used to dissolve artemisinins and partner drugs, respectively. Since the indicator ranges of the dipsticks for artemisinin, ATS, and DHA were reasonably low (in ng range), the sample stocks can be easily diluted in water to the sensitivity range of the samples. The dipstick was further tested for specificity to the artemisinin-related drugs, while it did not show detectable cross reactivity to other commonly used anti-malarials at 5,000 ng mL^-1^, suggesting that the partner drugs in the artemisinin-based combinations should not affect the performance of the dipsticks for analysing ACT samples.

This is a prototype dipstick and further improvements are needed for their use at the point of care. First, the mAb 3D_8_2G_7_ used to develop the dipsticks showed very limited cross reactivity to ATM. While in ELISA the IC_50_ of ATM was 6620 ng mL^-1^, the dipsticks did not show visible changes even at the maximum water solubility concentration of ATM, and thus are not suitable for ATM detection. The reason for the lack of cross reactivity with ATM is not clear, since all artemisinins have a similar structure opposite of position 12, where the carrier protein was conjugated to produce the immunogen. Therefore, this underscores the need to develop specific antibodies against ATM for the purpose of ATM detection. Second, the dipstick shows cross reactivity with artemisinin, ATS, and DHA. While this could be an advantage if the purpose is to use one dipstick format for detecting any of these three artemisinins, the development of specific mAbs is required if one wants to specifically detect each of the artemisinin derivatives. Third, further improvements are required for the stability of the dipsticks. Since these dipsticks were individually wrapped and sealed and humidity should not affect their performance, the impact of storage was tested at different temperatures. The results showed a gradual loss in the sensitivity of the dipstick over time. Therefore, for the current dipstick format, their sensitivity after storage needs to be re-evaluated and calibrated using standard artemisinin derivatives. Finally, the ultimate goal of this study is to deploy this convenient method as a point-of-care quality control device of artemisinin-based drugs. The current cost for the production of one dipstick is estimated to be 0.1-0.2 USD, which should have a promising market potential. As the currently used MiniLab tests for quality control of anti-malarials have been reported to detect only grossly substandard and counterfeit samples, the low indicator ranges of this dipstick assay for several artemisinin-based drugs may offer a solution to detect substandard and degraded artemisinins.

## Conclusions

A new selected mAb 3D_8_2G_7_ with high avidity and broad cross reactivity for artemisinins was used to develop and optimize a dipstick immunoassay for qualitative and semi-quantitative analysis of ATS and DHA in anti-malarial drugs. The indicator ranges of the dipsticks for ATS and DHA were 100-200 and 200-500 ng mL^-1^, respectively. The semi-quantitative analysis of ATS and DHA in commercial drugs and raw drug materials with the dipsticks produced result agreeable with those determined by HPLC, indicated the developed dipstick was reliable for semi-quantitation of artemisinin-based drugs. Specificity test of the artemisinins at a concentration of 5,000 ng mL^-1^ observed no colour inhibition on the test lines, which proved accurate performance of the dipsticks for analysing ACT samples. Storage test showed that the indicator range for artemisinins remained unchanged after a week at 37°C, but increased four-folds after six months of storage at 4°C or ambient temperature. Thus, the dipstick has the potential to be developed as a point-of-care device for identifying substandard and counterfeit ATS- and DHA-containing anti-malarial drugs.

## Abbreviations

ACT: Artemisinin-based combination therapy; APIs: Active pharmaceutical ingredients; ATM: Artemether; ATS: Artesunate; BSA: Bovine serum albumin; DHA: Dihydroartemisinin; DMEM: Dulbecco’s modified Eagle’s medium; FBS: Fetal bovine serum; HPLC: High performance liquid chromatography; icELISA: Indirect competitive ELISA; iELISA: Indirect ELISA; mAb: Monoclonal antibody; OVA: Ovalbumin; PVC: Polyvinyl chloride.

## Competing interests

The authors declare that they have no competing interests.

## Authors’ contributions

LSH, TGN and BMW conceived of the study, designed the experiments and analysed the data. LSH and TGN performed the experiments. LSH, TGN, YLC, SQG, WZ, RZ, GYT and LWC contributed reagents, materials and analysis tools. LSH wrote the paper. All authors read and approved the final manuscript.

## Supplementary Material

Additional file 1: Table S1 Reagents used in this study. Click here for file
