# Supplementary material for: Development of a colloidal gold-based lateral flow dipstick immunoassay for rapid qualitative and semi-quantitative analysis of artesunate and dihydroartemisinin
Source: Malar J. 2014 Mar 31;13:127. doi: 10.1186/1475-2875-13-127 (PMC4113134; doi:10.1186/1475-2875-13-127)
Supplement: Additional file 1: Table S1 — Reagents used in this study. [file 1475-2875-13-127-S1.doc]

**Additional file 1. Reagents used in this study.**

| **Reagents** | **Source** |
| --- | --- |
| ART | National Institute for the Control of Pharmaceutical and Biological Products (Beijing, China) |
| ATS |
| DHA |
| ATM |
| Quinine | J&K Chemical (Beijing, China) |
| Primaquine phosphate |
| Amodiaquine |
| Chloroquine diphosphate salt | Sigma-Aldrich (St Louis, MO, USA) |
| Pyrimethamine |
| Lumefantrine |
| Piperaquine tetraphosphate tetrahydrate |
| Pyronaridine tetraphosphate |
| Penicillin |
| Streptomycin |
| L-glutamine |
| APIs of ATS | Online stores |
| APIs of DHA |
| Cell culture medium (DMEM) | Gibco BRL (PaisLey, Scotland) |
| FBS |
| Chromatographic grade acetonitrile | Fisher Scientific (USA) |
| GBA^+^ buffer | Jieyi Biotechnology Co., Ltd. (Shanghai, China) |
| Sartorius CN140 membranes |
| Ahlstrom 8964 glass fiber |
| GL-b0701 sample pad |
| H5076 absorbent pad |
| DB-6 PVC backing |
| A-9 plastic housing |
| Aluminum foil pouch |
| Goat anti-mouse IgG |
